# Identification of *TH* Variants in Chinese Dopa-Responsive Dystonia Patients and Long-Term Outcomes

**DOI:** 10.3389/fneur.2021.644910

**Published:** 2021-05-12

**Authors:** Xin-yao Li, Ying-mai Yang, Li-bo Li, Meng-yu Zhang, Yang-yu Huang, Jie Wang, Lin Wang, Xin-hua Wan

**Affiliations:** Department of Neurology, Peking Union Medical College Hospital, Chinese Academy of Medical Sciences, Beijing, China

**Keywords:** dopa-responsive dystonia, tyrosine hydroxylase deficiency, levodopa, long-term outcomes, meta-iodobenzylguanidine

## Abstract

**Background:** Dopa-responsive dystonia (DRD) is a movement disorder that is highly clinically and genetically heterogeneous. Our study summarizes clinical characteristics and long-term outcomes in patients with dopa-responsive dystonia with the aim of obtaining further knowledge on this disorder.

**Methods:** Patients who met DRD genetic diagnostic criteria through whole-exome sequencing and took levodopa for over 3 years were included in our study. Detailed information was collected on these patients, including family history, age at onset, age and dosage at starting levodopa, current medication and dosage, levodopa duration, diurnal fluctuation, and other clinical features. The Burke–Fahn–Marsden Dystonia Rating Scale-Motor (BFMDRS-M) score was used to evaluate patients' dystonia and variation after levodopa. According to the long-term outcomes, patients were further graded as good (dystonia improved by more than 50% after levodopa, and no further motor symptoms appeared) and poor (dystonia improved by <50% after levodopa, or new motor symptoms appeared).

**Results:** A total of 20 DRD patients were included (11 with *GCH1* variants, 9 with *TH* variants). During long-term levodopa treatment, three patients with *TH* variants (3/20, 15%) developed motor symptoms, including body jerks and paroxysmal symptoms, and responded well to increasing levodopa doses. The patient with homozygous mutation c.1481C>T/p. Thr494Met harbored more serious symptoms and poor response to levodopa and showed decreased cardiac uptake in MIBG.

**Conclusions:** Most DRD patients showed satisfactory treatment outcomes after long-term levodopa, whereas few patients with *TH* variants presented motor symptoms, which is considered to be related to dopamine insufficiency. For patients with motor symptoms after long-term levodopa, increasing the dose slowly might be helpful to relieve symptoms.

## Background

Dopa-responsive dystonia (DRD) comprises a series of highly clinically and genetically heterogeneous disorders, among which GTP-CH-I deficiency is the most well-known disorder. However, DRD can still be caused by defects in other enzymes that are involved in dopamine biosynthesis, such as tyrosine hydroxylase [TH, encoded by *TH* gene (OMIM 191290)], sepiapterin reductase [SR, encoded by the *SPR* gene (OMIM 182125)], pyruvoyl-tetrahydropterin synthase [PTPS, encoded by the *PTS* gene (OMIM 612719)], pterin-4a-carbinolamine dehydratase [PCD, encoded by the *PCBD* gene (OMIM 126090)], and dihydropteridine reductase [DHPR, encoded by the *QDPR* gene (OMIM 612676)]. Tyrosine hydroxylase (TH) is an iron-containing monooxygenase that catalyzes the first and rate-limiting step in the biosynthesis of catecholamines (dopamine, noradrenaline, and adrenaline) with its essential cofactor tetrahydrobiopterin (BH4). Tyrosine hydroxylase deficiency (THD), an autosomal recessive hereditary dopa-responsive dystonia (DRD), is caused by mutations in the tyrosine hydroxylase (*TH*) gene at chromosome 11p15.5 (1). Based on the largest clinical phenotypic research to date on patients with TH deficiency, it is rated as type A (progressive hypokinetic-rigid syndrome with dystonia) and type B (complex encephalopathy) (1). Compared to type B, type A is considered to exhibit a more satisfactory efficacy to low-dose levodopa and less residual motor or cognitive impairment. To our knowledge, little has been reported on long-term outcomes in DRD patients, especially for patients with TH deficiency; one subject in 36 TH deficiency patients developed dyskinesia after levodopa for 13 years (1), and another two siblings who initiated from lower limbs showed a sustained response to levodopa for over 35 years (2). In this paper, we present long-term outcomes in TH deficiency patients with the aim of obtaining further knowledge on this rare disease.

## Patients and Methods

The study was carried out in a cohort of dystonia patients who visited the Movement Disorders Clinic in the Department of Neurology of Peking Union Medical College Hospital from February 2016 to November 2019. All the subjects were genetically diagnosed with DRD through whole-exome sequencing (WES) (Supplementary Material). Genetic criteria refer to patients whose genetic analysis shows mutation of the *GCH1, TH, SPR, PTS*, PCBD, or *QDPR* genes. On admission, the clinical data were carefully collected, including family history, age at onset, age and dosage at starting levodopa, duration of levodopa, current medication and dosage, diurnal fluctuation, and other clinical features. Patients who met genetic criteria and took levodopa for more than 3 years were further included in our study. Written informed consent was obtained from all participating individuals or their legal guardians.

A total of 20 subjects (13 females, 7 males) were identified. Eleven patients had *GCH-1* mutations, and nine patients had *TH* mutations. The Burke–Fahn–Marsden Dystonia Rating Scale-Motor (BFMDRS-M) score was used to evaluate patients' dystonia and variation after levodopa. Long-term outcomes were scored as follows: (a) good, dystonia improved by more than 50% after levodopa, and no further motor symptoms appeared; (b) poor, dystonia improved by <50% after levodopa, or new motor symptoms appeared. According to the classification standard, 17 patients showed a good response to levodopa, whereas three patients with *TH* variants presented a poor response.

## Results

The clinical features of 20 DRD patients are summarized in Tables 1, 2. Nine patients with *TH* variants are identified. Among these patients, three patients show unsatisfactory outcomes.

**Table 1:** Clinical features in TH deficiency patients.

**Patients with** ***TH*** **variants**									
**Patient**	**1**	**2**	**3**	**4**	**5**	**6**	**7**	**8**	**9**
cDNA	c.1481C>T	c.694C>T, c.734G>T	c.698G>A, c.679G>C	c.698G>A	c.457C>T, c.739G>A	c.694C>T,c.739G>A	c.734G>T c.739G>T	c.1321C>T, c.1495G>A	c.457C>T, c.1321C>T
Genotype^a^	p. Thr494Met (Homozygous)	p. Gln232Ter,p. Arg245Met	p. Arg233His, p. Asp227His	p. Arg233His(Homozygous)	p. Arg153Ter, p. Gly247Ser	p. Gln232Ter,p. Gly247Ser	p. Arg245Met, p. Gly247Ser	p. Arg441Trp,p. Val499Met;	p. Arg153Ter, p. Arg441Trp
Age (y), sex	34, F	39, F	26, M	28, M	35, M	13, M	26, F	33, F	47, F
Family history	N	N	N	N	N	N	N	N	N
Age at onset (y)	24	7	6	3	6	6	17	5	21
Site of onset	BLL	BLL	BLL	RLL	BLL	BLL	LLL	RLL	Neck, trunk
Age (y) at starting LD	25	19	10	24	27	10	20	16	25
Initial LD dosage^*^ (mg)	LD/BE 200/50	LD/BE 50/12.5	LD/BE 150/37.5	LD/BE 100/25	LD/BE 100/25	LD/BE 50/12.5	LD/BE 100/25	LD/BE 100/25	LD/BE 200/50
Current drugs and dosage^*^ (mg)	LD/BE 250/62.5; THP 4; CLZ 0.5	LD/BE 150/37.5	LD/BE 200/50; THP 4	LD/BE 100/25; THP 4; BLF 20	LD/BE 100/25; SEL 15; BLF 30; THP 3	LD/BE 50/12.5	LD/BE 100/25	LD/BE 100/25	LD/CBD 200/50; BLF 20
Clinical features									
Muscular hypotonia	N	N	N	N	N	N	N	N	N
Oculogyric crises	N	Y	N	N	N	N	N	N	N
Myoclonus	Y	N	N	N	N	N	N	N	N
Cognitive dysfunction	N	N	Y (Mild)	N	N	N	N	N	N
Tremor	N	N	N	N	N	N	N	N	N
Rigidity	N	N	Y	N	N	N	N	N	N
Dystonia	Y	N	Y	Y	Y	Y	N	N	Y
Diurnal fluctuation	N	Y	Y	Y	N	N	N	Y	N
Duration of LD (y)	9	20	16	4	8	3	6	17	22
LD response and current symptoms	Poor; Body jerks	Poor;Oculogyric crisis	Poor; Paroxysmal symptoms	Good; Wide-based gait	Good; Wide-based gait, anxiety	Good; Not reported	Good; Not reported	Good; Not reported	Good; Not reported
Brain MRI	Normal	Normal	Normal	Normal	Normal	Normal	Normal	Normal	Normal
DAT-PET	Normal	Normal	−	−	−	−	−	−	Normal
MIBG	decreased cardiac uptake	Normal	−	−	−	−	−	−	Normal
Cerebrospinal fluid									
HVA	89.531	39.27	−	−	−	−	−	−	39.348
5-HIAA	61.868	59.324	−	−	−	−	−	−	25.016
HVA/5-HIAA	1.447	0.66	−	−	−	−	−	−	1.573
MHPG	15.536	24.249	−	−	−	−	−	−	29.996
3-OMD	175.47	6.959	−	−	−	−	−	−	6.81

a *Based on GenBank: NM_199292.3*.

* *A daily dose of medication*.

*F, female; M, male; Y, yes; N, no; y, year; BLL, bilateral lower limb; RLL, right lower limb; LLL, left lower limb; LD/BE, levodopa/benserazide; LD, levodopa; CBD, carbidopa; THP, trihexyphenidyl; CLZ, clonazepam; BLF, baclofen; SEL, selegiline; DAT-PET, dopamine transporter positron emission computed tomography; MIBG, metaiiodo-benzylguanidine; HVA, homovanillic acid (normal 145–432 nmol/L); 5-HIAA, 5-hydroxyindole acetic acid (normal 67-140 nmol/L); HVA/5-HIAA (normal 1.04–4.90); MHPG, 3-methoxy-4-hydroxyphenethyleneglycol (normal 29–64 nmol/L); 3-OMD, 3-O-methyldopa (normal <60 nmol/L)*.

**Table 2:** Clinical features of patients with *GCH1* variants.

	**Patients with** ***GCH1*** **variants**															
**Patient**	**cDNA**	**Genotype^a^**	**Sex**	**Age (y)**	**Family history**	**Age at onset (y)**	**Site of onset**	**Onset symptoms**	**Associated symptoms**	**Age (y) at starting LD**	Initial LD dosage^*^ (mg)	**Duration (y) of LD**	Current LD dosage^*^ (mg)	**Diurnalfluctuation**	**Brain MRI**	**Outcomes**
10	c.557C>A	p. Thr186Lys	F	31	N	6	BLL	Dystonia	−	24	100	7	150	Y	Normal	Good
11	c.328C>T	p. Gln110Ter	M	24	N	12	BLL	Dystonia	−	20	100	4	100	Y	Normal	Good
12	c.693G>C	p. Leu231Phe	M	26	N	8	LL	Dystonia	−	16	50	10	200	N	Normal	Good
13	c.551G>A	p. Arg184His	M	21	N	6	BLL	Dystonia	Parkinsonism	10	100	11	250	Y	Normal	Good
14	c.632T>C	p. Met211Thr	F	23	N	19	LUL	Dystonia	−	20	50	3	50	Y	Normal	Good
15	c.614T>A	p. Val205Glu	F	32	Y	29	BLL	Dystonia	−	29	200	3	250	Y	Normal	Good
16	c.263G>A	p. Arg88Gln	F	23	N	11	LL	Dystonia	Parkinsonism	17	100	6	250	Y	Normal	Good
17	c.316A>G	p. Thr106Ala	F	46	N	42	UL	Dystonia	Tremor	43	250	3	250	N	Normal	Good
18	c.327C>G	p. Tyr109Ter	F	29	N	16	LL	Dystonia	−	20	150	9	250	Y	Normal	Good
19	c.638_641del	p. Lys213Ilefs^*^5	F	44	N	26	Neck	Dystonia	−	32	200	12	250	N	Normal	Good
20	c.514delG	p. Val172Ter	F	13	N	10	Neck	Dystonia	−	10	50	3	100	Y	Normal	Good

a *Based on GenBank: NM_000161.3*.

* *A daily dose of medication*.

*F, female; M, male; Y, yes; N, no; y, year; LL, lower limb; UL, upper limb; BLL, bilateral lower limb; LUL, left upper limb; LD, levodopa*.

### Genetic Detection

*TH* variants were detected by WES, including seven missense variants and two nonsense variants, mainly located in *TH* exons 4, 6, 7, 13, and 14. All variants are rated as pathogenic or likely pathogenic according to the ACMG criterion (3). The father of patient 8 was deceased, the parents of two patients (patients 2 and 4) were not available for genetic testing, and segregation analysis of these three patients was incomplete. Variants in the other six patients were confirmed to be inherited from their mother or father by segregation analysis. Three variants are novel (c.679G>C/p. Asp227His, c.1321C>T/p. Arg441Trp, c.1495G>A/p. Val499Met), whereas another six have been reported previously in other subjects with TH deficiency (4–8).

Four variants (p. Asp227His, p. Arg233His, p. Arg245Met, p. Gly247Ser) are located in exon 6 within the catalytic domain. The p. Asp227His variant is predicted to be disease causing (CADD Phred 27.8) and affects a highly conserved amino acid. Substitution from aspartic acid to glycine at position 227 is predicted to cause complete loss of tyrosine hydroxylase activity (9). The substituted arginine-233 residue is a highly conserved protein in various eukaryotic species and is scored 34 by CADD. Functional analysis revealed that the residual activity of this variant protein was reduced by more than 80% (10). p. Arg245Met is absent in GnomAD, with a CADD phred 26.1. It is predicted to be damaging by multiple prediction tools (PolyPhen2, SIFT, and MutationTaster). The p. Gly247Ser variant affects a highly conserved amino acid and has a CADD Phred score of 26.5. This mutant leads to the loss of ~50% of tyrosine hydroxylase activity (11).

The p. Arg441Trp mutation causes substitution of a moderately conserved arginine of the TH protein and is predicted to be deleterious *in silico* (CADD Phred 28.2). It has been reported that substitution from arginine to proline leads to complete loss of enzyme activity (11).

p. Thr494Met and p. Val499Met is located within the oligomerization domain, which affects highly and moderately conserved amino acids. Both variants have CADD scores higher than 23 and are predicted to be deleterious by PolyPhen2, SIFT, and MutationTaster.

### Clinical Characteristics

In patients with TH deficiency, the ratio of females to males was 5:4 (5/4). The median age of onset was 10.6 years old (ranging from 3 to 24 years old). Of these, the onset age of two patients (patients 1 and 9) was over 20 years old. Disease duration varied from 7 to 32 years (average 20.7 ± 9.6 years). Eight patients were onset from lower limbs, presenting as tiptoe walking or foot inturning. Another one was initiated from trunk and neck. The median age of starting levodopa was 19.6 years (ranging from 10 to 27 years). The median duration of levodopa use was 11.7 years (ranging from 3 to 22 years).

During the long-term course, three patients (Patients 1, 2, and 3) showed motor symptoms, including body jerks and paroxysmal symptoms. One patient (Patient 5) showed non-motor symptoms and was in a state of anxiety. In addition, although patient 4 and patient 5 showed satisfactory treatment outcomes, both had some residual symptoms, showing splayfoot deformity (Videos 1, 2 in Supplementary Material). During pregnancy, diurnal variation was extremely evident in patient 2, who maintained a daily dose of levodopa/benserazide 50/12.5 mg. On the other hand, due to withdrawal of levodopa, symptoms were aggravated in patient 8 when she was pregnant, and she was forced to terminate pregnancy. All patients underwent brain magnetic resonance imaging, showing no abnormalities. Three patients conducted dopamine transporter positron emission tomography (DAT PET), meta-iodobenzylguanidine (MIBG), and biochemical analysis of the cerebrospinal fluid (CSF); the results are shown in Table 1.

### Three Patients With Poor Treatment Outcomes

**Figure 1:**
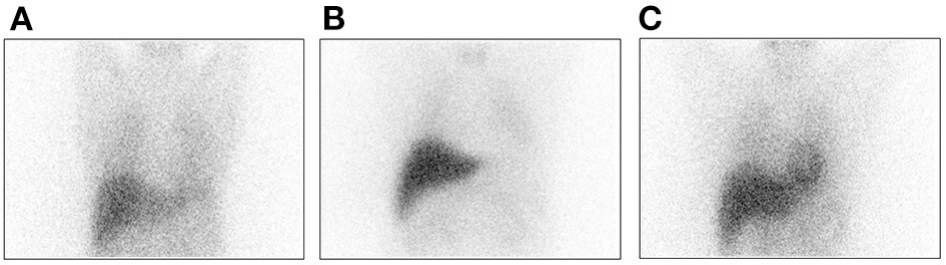
MIBG imaging of patient 1 indicates decreased cardiac uptake **(A)** Patient 1; **(B)** PD patient; and **(C)** Normal control subject.

Patients 1 through 3 developed motor symptoms after long-term levodopa. Body jerks were extremely evident in patient 1, leading to severe limitations in daily activities (Video 3 in Supplementary Material). DAT-PET was normal, and MIBG showed decreased cardiac uptake (Figure 1). Paroxysmal upward gaze in patient 2 occurred suddenly and lasted for 2–4 h (Video 4 in Supplementary Material). The attack occurred ~4–5 times a week. When it appeared, the patient felt mental slowness, palpitations, and upper limb rigidity. It occurred frequently and lasted even longer when she was tired or got cold. Difficulty in daily work was also reported. Patient 3 showed paroxysmal symptoms. When it attacked, the head turned to the right, with the right hand raised, lasting for ~20–30 min (Video 5 in Supplementary Material). During the attack, he was in clear consciousness, with a normal 24-h electroencephalogram. At present, the attack occurred one to three times per year.

All three patients were treated with levodopa/benserazide (1.00:0.25); the starting doses were 200/50, 50/12.5, and 150/37.5 mg/d, and the initial dose was maintained for 9, 20, and 16 years, respectively. According to individual tolerance, the dose was increased slowly within 2–4 weeks. The doses in patients 2 and 3 were increased up to 150/37.5 and 200/50 mg/d, respectively. The frequency of attacks in these two patients decreased. Because patient 1 was unable to tolerate the side effects of the dosage increase, the dose was only increased up to 250/62.5 mg/d, and clonazepam (0.5 mg/d) was introduced, with mild ameliorated body jerks, but still had severe limitations in daily activities.

## Discussion

In 20 DRD patients, nine patients with *TH* variants were detected, including seven with compound heterozygosity and two with homozygous mutations. A total of 72.2% (13/18) of variants were located in the catalytic domain, 16.7% (3/18) were in the oligomerization domain, and another 11.1% (2/18) were within the regulatory domain. The most common mutations were p. Arg233His (mutation frequency is 21.4%) and p. Gly247Ser (mutation frequency is 21.4%), which was consistent with the results of another Chinese large-scale study that included 23 patients with TH deficiency (mutation frequency of p. Arg233His is 20.5%) (6). Therefore, we speculated that the variant, p. Arg233His, was the mutation hotspot in China.

Recessive hereditary dopa-responsive dystonia, tyrosine hydroxylase deficiency, has a far more complex phenotype than dominant GTP-CH-I deficiency, ranging from focal or generalized dystonia and parkinsonian symptoms (hypokinesia, rigidity of extremities, tremor) to oculogyric crises, myoclonus, intellectual impairment, and autonomic dysfunction (12). According to the TH deficiency classification, all of our patients should be classified as type A. Patient 1 developed prominent motor symptoms in the long-term treatment with levodopa. The mutation, c.1481C>T/p. Thr494Met, located within the oligomerization domain, has been reported in several other subjects with TH deficiency (6, 7, 13). Mutations varied from homozygous and compound heterozygous to heterozygous mutations (Table 3). Of note, the disease features of the patient reported by Katus and Frucht (7) were extremely similar to those of our patient 1. Both were homozygous for the p. Thr494Met mutation and presented unsatisfactory responses to levodopa over the long-term course. This variant was even reported as one single heterozygous and segregates with an infantile-onset, hypokinetic-rigid syndrome with dystonia (6).

**Table 3:** Clinical features of TH-deficient patients carrying the p. Thr494Met mutation reported to date.

**Clinical features**	**Our patient 1**	**Patients [Swaans et al**. **(****13****)]**		**Patient B [Katus and Frucht (7)]**	**Patient C [Chen et al. (6)]**
		**Patient A**	**Patient A1**		
Genotype^a^	p. Thr494Met, homozygous	p. Thr494Met, compound heterozygous		p. Thr494Met, homozygous	p. Thr494Met, heterozygous
Family history	N	Y	Y	N	N
Age, sex	34 y, F	>35 y, M	>39 y, M	31 y, M	>2 y, M
Age at onset	24 y	2 y	5 y	18 y	4.5 m
Site of onset	BLL	LL	UL	RUL	Generalized
Current symptoms	Generalized dystonia	−	−	Segmental dystonia (neck, trunk)	−
Other illness	Thalassaemia	−		Malaria	−
Sensory tricks	Y	−		Y	−
Diurnal variation	Unremarkable	−		N	Y
Age at starting LD	25 y	>5 y	>9 y	>21 y	>4.5 m
Trial of medication	LD; THP; ELD; CLZ; PRA	LD; CBD		THP; CBD/LD	LD
Current medication^*^ (mg)and efficiency	LD/BE 250/62.5 mg; THP 4 mg; CLZ 0.5 mg. Poor response to LD	Low-dose LD. Response to LD for more than 30 years		CBD/LD 45/450 mg; THP, 4 mg. Without a complete response to LD	LD 4.6 mg/kg/d.Response well to LD

a *Based on GenBank: NM_199292.3*.

* *A daily dose of medication*.

*F, female; M, male; y, year; m, month; Y, yes; N, no; LL, lower limb; UL, upper limb; BLL, bilateral lower limb; RUL, right upper limb; THP, trihexyphenidyl; LD, levodopa; CBD, carbidopa; ELD, Eldepryl; CLZ, clonazepam; PRA, Pramipexole; BE, benserazide*.

Human TH includes an N-terminal regulatory domain, a catalytic domain, and a C-terminal tetramerization domain. Mutations in active sites or the tetramerization domain are predicted to exert a greater influence on TH protein function (9, 14). The oligomerization domain is formed by two β-strands and one α-helix. It is suggested that alterations of the C-terminal zipper sequence not only abolish tetramer formation but also decrease the TH enzyme activity (11). The β-strands and the loop preceding the α-helix are predicted to complete the rest of the formation of TH tetramers and promote oligomerization (14). Indeed, activity assays revealed that the missense variant (D467G) located in the tetramerization domain was more damaging than the variants within the regulatory and catalytic domains, which retained only 5–7% TH activity compared to the WT enzyme (15). Variants in this region, especially homozygous mutations, are predicted to exert a greater impact on TH enzyme activity. Disruption of TH protein might cause deficiency of noradrenaline in the sympathetic nerves, leading to alterations in the cardiovascular systems (16, 17), which might explain the decreased cardiac uptake in MIBG.

Poor outcomes in three patients were considered to be related to dopamine insufficiency. Animal studies have shown that aging not only reduces TH protein in the substantia nigra (SN) (18) but also reduces nigral TH activity (19). Loss of nigral total TH protein was up to 40% in the SN in 30-month rats compared to 12-month rats (20). The postulated mechanism for the reduction in TH activity is considered to be related to site-specific phosphorylation (Ser19, Ser31, and Ser40) in the central nervous system (21). It was suggested that phosphorylation at Ser31 increases TH activity (21), whereas a 30% decrease in TH phosphorylation at Ser31 was observed in the older rat group (20). Age-related reduction in TH phosphorylation leads to the attenuation of enzymatic activity. Under the premise of TH enzyme activity insufficiency, age-related susceptibility made initial low-dose levodopa no longer meet the requirements of dopamine metabolism; thus, motor symptoms appeared.

It was suggested that the accurate clinical diagnosis of TH deficiency should be based on mutation analysis of the *TH* gene and central catecholamine deficiency, that is, low HVA, low MHPG, low HVA/HIAA ratio, and normal 5-HIAA in CSF (1, 22). The HVA levels and HVA/5-HIAA ratio were also considered to be correlated with the phenotypes and treatment outcomes (1, 4). However, the CSF neurotransmitter metabolites in our adult patients with TH deficiency seemed to present different results. All three patients showed low 5-HIAA, whereas the HVA/HIAA ratio was normal in two patients. It has been suggested that reference values of HVA and 5-HIAA in CSF decrease with age (22). Even if levodopa was discontinued before the collection of CSF, we still needed to take into account the long-term effect of levodopa on dopamine metabolism in the body.

Norepinephrine (NE), an essential catecholamine, is correlated with many brain functions, such as anxiety, depression, attention, and memory (23). NE deficiency due to *TH* variants leads to numerous brain dysfunctions, especially anxiety and depression. It might explain the emotional problem in patient 5. Residual walking issues in patient 4 and patient 5 were considered to be related to the long duration from onset to taking levodopa (~21 years), which caused irreversible skeletal malformations. Therefore, all hereditary dystonia patients with childhood onset should be recommended for experimental treatment to make earlier clinical diagnoses and initiate etiological therapy.

Two women (patient 2 and patient 8) had experienced pregnancy. During pregnancy, one fetus was maintained with a low dose of levodopa, and the fetus was born safely. However, the other woman underwent levodopa withdrawal, leading to symptoms that were intolerable, and was forced to terminate her pregnancy. Therefore, we considered that levodopa should be maintained at a low dosage (~50 mg) during pregnancy in TH-deficient patients.

In conclusion, our study further deepens the understanding of TH deficiency. Patients with *TH* variants might develop motor symptoms after long-term levodopa; increasing the dose slowly might be helpful to relieve symptoms.

## Data Availability Statement

Publicly available datasets were analyzed in this study. This data can be found here: National Center for Biotechnology Information (NCBI) GenBank, https://www.ncbi.nlm.nih.gov/genbank/, NM_199292.3, NM_000161.3, NM_003124.5.

## Ethics Statement

The studies involving human participants were reviewed and approved by the ethics committee of Peking Union Medical College Hospital. Written informed consent to participate in this study was provided by the participants' legal guardian/next of kin. Written informed consent was obtained from the individual(s), and minor(s)' legal guardian/next of kin, for the publication of any potentially identifiable images or data included in this article.

## Author Contributions

X-yL: data acquisition, statistical analysis, and writing of the first draft. LW and X-hW: study concept and design and critical revision of manuscript. Y-mY, L-bL, M-yZ, Y-yH, and JW: interpretation of data and revision of manuscript. All authors contributed to the article and approved the submitted version.

## Conflict of Interest

The authors declare that the research was conducted in the absence of any commercial or financial relationships that could be construed as a potential conflict of interest.
